# African swine fever virus alters soil microbial biomass and biodiversity: Evidence from experimental soil systems

**DOI:** 10.14202/vetworld.2026.1984-1998

**Published:** 2026-05-16

**Authors:** Zaven Karalyan, Anahit Sedrakyan, Karine Arakelova, Magdalina Zakharyan, Shoghik Hakobyan, Sona Hakobyan, Aida Avetisyan, Nane Bayramyan, Hranush Arzumanyan, Vahagn Gevorgyan, Tigranuhi Vardanyan, Bagrat Baghdasaryan, Alexander Karalyan, Lina Hakobyan, Arpine Poghosyan, Liana Abroyan, Elena Karalova, Henry Voskanyan, Zara Semerjyan, Elina Arakelova, Hranush Avagyan

**Affiliations:** 1 Laboratory of Cell Biology and Virology, Institute of Molecular Biology NAS RA, Yerevan, Armenia; 2 Department of Medical Biology, Yerevan State Medical University after Mkhitar Heratsi, Yerevan, Armenia; 3 Laboratory of Microbial Genomics, Institute of Molecular Biology NAS RA, Yerevan, Armenia; 4 Viral Ecology Research Group, Institute of Molecular Biology NAS RA, Yerevan, Armenia; 5 Experimental Laboratory, Yerevan State Medical University after M. Heratsi, Yerevan, Armenia; 6 Institute of Biology, Yerevan State University, Yerevan, Armenia; 7 Scientific Center for Risk Assessment and Analysis in Food Safety Area, Yerevan, Armenia

**Keywords:** African swine fever virus, biodiversity, environmental virology, microbial biomass, quantitative real-time polymerase chain reaction, soil ecology, soil microbiome, viral ecology

## Abstract

**Background and Aim:**

African swine fever virus (ASFV) has expanded beyond its traditional ecological niches, raising concerns not only for animal health but also for environmental sustainability. While extensive research has focused on its persistence and transmission, little is known about its ecological effects in soil systems. This study aimed to investigate the influence of ASFV on soil microbial biomass, biodiversity, and associated ecological parameters.

**Materials and Methods:**

Eighteen anthrosol soil samples collected from agricultural regions of Armenia were subjected to controlled experimental conditions. Soil samples were treated with active ASFV (aASFV), inactivated ASFV (iASFV), and mock controls. Physicochemical properties, including pH and moisture content, were assessed. Microbial biomass was evaluated through soil protein quantification and viral nucleic acid (DNA and RNA) measurements. Microbial diversity was analyzed by enumerating culturable bacteria and fungi using selective media. Dissolved oxygen levels were measured to assess microbial activity. Quantitative real-time polymerase chain reaction was employed to evaluate viral genome dynamics and transcriptional activity. Statistical analyses were performed to determine correlations among measured variables.

**Results:**

ASFV exposure resulted in a general reduction in total microbial biomass, as evidenced by decreased soil protein content and viral nucleic acid concentrations in most samples. In contrast, microbial diversity, particularly among bacterial and fungal populations, showed an increasing trend, suggesting a restructuring of the microbial community. Active ASFV induced greater changes compared to the inactivated virus. A significant positive correlation was observed between protein content and microbial indicators, while a negative correlation was noted between oxygen levels and nucleic acid content. Viral transcriptional activity was detected in selected samples, with no evidence of complete viral replication. Limited detection of giant viruses suggested potential but inconclusive ecological interactions.

**Conclusion:**

ASFV alters soil ecosystems through complex, multidirectional effects, characterized by reduced biomass and increased microbial diversity. These findings indicate that ASFV may indirectly influence soil ecological processes, even in the absence of active replication. The study highlights the importance of incorporating environmental perspectives into ASFV research and provides a foundation for future investigations on virus–soil–microbiome interactions.

## INTRODUCTION

The African swine fever virus (ASFV) has demonstrated a remarkable capacity to adapt and persist in regions lacking its primary natural reservoir, the tick *Ornithodoros moubata*. Furthermore, ASFV can persist and sustain transmission cycles in the absence of African wild suids, such as bushpigs and warthogs, which typically sustain viremia without developing clinical disease and thus serve as natural reservoirs for the virus [1, 2]. This ecological flexibility highlights ASFV’s ability to establish itself in novel environments beyond its traditional epidemiological boundaries.

Evidence regarding the broader ecological consequences of ASFV remains limited. However, one of the few studies investigating indirect ecological effects reported that ASFV outbreaks affected raven populations [3]. Additionally, ASFV has been shown to act as a potential nutritional source for ciliates [4]. These findings suggest that ASFV may participate in ecological interactions extending beyond classical host–pathogen dynamics. Such interactions raise the possibility that ASFV influences ecosystem processes indirectly by interacting with non-host organisms.

The soil environment plays a fundamental role in sustaining terrestrial ecosystems by providing essential services, including nutrient cycling, support for plant growth, water storage and purification, and the maintenance of biodiversity. Soil ecosystems host a vast diversity of organisms, particularly microorganisms that drive key biochemical and ecological processes. Healthy soils are therefore critical for agricultural productivity, climate regulation through carbon sequestration, and the preservation of ecosystem stability.

The environmental fate of animal viruses in soil has been described previously [5], and similar mechanisms are likely to apply to ASFV. Potential pathways for ASFV introduction into soil include contamination through grazing by infected pigs and the deposition and decomposition of carcasses of infected animals, which can serve as significant sources of environmental viral load [6]. Most existing studies addressing ASFV in soil environments have primarily focused on viral persistence and survival duration [7, 8] or on strategies for virus inactivation and elimination [9, 10].

Environmental factors such as pollutants, elevated temperatures, acidic pH, solar radiation, and soil structure are known to influence viral survivability in soil matrixes [5]. Notably, ASFV has been reported to remain stable under a wide range of conditions, particularly in non-acidic soils [7]. Despite these advances, research on ASFV in environmental contexts has largely focused on persistence and inactivation dynamics, with comparatively little attention to its potential ecological effects on soil microbial communities.

Viruses are increasingly recognized as important ecological drivers in diverse ecosystems. They play critical roles in regulating microbial biomass, shaping community diversity, and influencing nutrient cycling processes. In soil ecosystems, viruses contribute to the structuring of microbial communities by affecting their composition, abundance, and functional activity [11, 12]. These roles suggest that ASFV, despite being primarily an animal pathogen, may also exert indirect ecological effects within soil environments.

Although ASFV has been extensively studied in the context of transmission dynamics, host-pathogen interactions, and environmental persistence, its ecological impact on soil microbial systems remains poorly understood. Existing studies have predominantly focused on survival kinetics and inactivation strategies, leaving a critical gap in understanding how ASFV influences microbial biomass, biodiversity, and ecosystem functioning. Moreover, no integrative studies have simultaneously examined molecular, microbiological, and physicochemical parameters to evaluate ASFV–soil interactions under controlled experimental conditions. This lack of multidimensional investigation limits the ability to interpret ASFV within a broader ecological and One Health framework, particularly regarding its indirect effects on non-host organisms and soil ecosystem processes.

The present study was designed to address these knowledge gaps by systematically evaluating the ecological effects of ASFV on soil microbial systems. Specifically, the study aimed to assess the impact of ASFV on key indicators of soil ecosystem function, including microbial biomass, biodiversity, and associated environmental parameters. To achieve this, both active ASFV (aASFV) and inactivated ASFV (iASFV). were introduced into controlled soil microcosms, allowing differentiation between potential replicative and non-replicative effects.

The study further aimed to quantify changes in soil microbial biomass using protein content and viral nucleic acid measurements, and to evaluate shifts in microbial diversity by enumerating culturable bacteria and fungi. In addition, the investigation sought to analyze ASFV genome dynamics and transcriptional activity using qPCR, alongside assessing physicochemical parameters such as DO, which reflect microbial metabolic activity. By integrating these approaches, the study aimed to determine whether the presence of ASFV is associated with measurable ecological shifts in soil systems.

Ultimately, this work aimed to test the hypothesis that ASFV can act as an indirect ecological modulator, influencing soil microbial communities and ecosystem processes even in the absence of productive viral replication. The findings are intended to expand the current understanding of ASFV beyond its role as a livestock pathogen, highlighting its potential environmental significance and contributing to a more comprehensive One Health perspective.

## MATERIALS AND METHODS

### Ethical approval

Soil sampling and experimental procedures were conducted in accordance with the environmental regulations of the Republic of Armenia. All sampling sites were located on agricultural lands and did not involve protected ecosystems, endangered species, or restricted areas; therefore, no specific environmental permits were required. The study design complied with national and institutional guidelines for environmental research and biosafety.

All laboratory procedures involving ASFV were performed in compliance with established biosafety regulations for handling high-consequence animal pathogens. Experimental work was conducted in a certified biosafety facility under controlled conditions, following standard operating procedures for containment, decontamination, and waste disposal. Personnel involved in the study were trained in biosafety practices, and appropriate personal protective equipment was used throughout all experimental procedures. Handling of infectious material, including preparation of viral inoculum and disposal of contaminated samples, was carried out in accordance with institutional biosafety guidelines to prevent environmental release and cross-contamination.

The study protocol was reviewed and approved by the Ethics Committee of the Institute of Molecular Biology, National Academy of Sciences of the Republic of Armenia (IRB No. 00004079, 2013; Protocol No. 5, May 25, 2018; and IRB No. 06042021/1, 2021). The committee confirmed that the study did not involve live animal experimentation or human subjects. Biological materials used for virus propagation were obtained from previously established laboratory sources, and no animals were sacrificed specifically for this study.

All experimental procedures, including soil handling and post-experimental processing, were performed under strict biosafety conditions. At the end of the experiment, all soil samples and biological materials were sterilized by autoclaving (121°C, 2 atm, 20 min) before disposal. These measures ensured compliance with institutional, national, and international standards for biosafety and environmental protection.

Soil samples were collected in accordance with the environmental regulations of the Republic of Armenia. All sampling sites were located on agricultural land and not within protected or restricted natural areas; therefore, no specific environmental permits were required for sample collection. Sampling activities did not involve protected species or ecosystems, and no ethical limitations affected the sample size. The studies were reviewed and approved by the Ethics Committee of the Institute of Molecular Biology NAS RA (IRB 00004079, 2013; Protocol N5, May 25, 2018). The study protocol was approved by the Ethics Committee of the Institute of Molecular Biology, NAS RA (IRB 06042021/1, 2021).

### Study period and location

The study was conducted from March to May 2023 at Institute of Moleucular Biology NAS RA.

### Soil sampling and pre-experimental preparation

**Figure 1:**
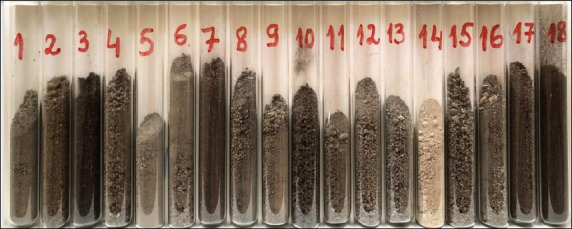
Appearance of soil samples used in the study, with corresponding pH values and geographic locations.

Eighteen anthrosol soil samples were collected from agricultural areas located in different regions of the Republic of Armenia. Soil was collected from the top layer (1 cm depth) using sterile stainless-steel tools to avoid cross-contamination. The sandy, coarse texture of the soils was determined using the standard field texture classification (hand-feel method). Types of soil samples are presented in Figure 1. Immediately after collection, samples were placed in sterile, airtight containers and transported to the laboratory under refrigeration.

Prior to analysis, soils were homogenized and sieved through a sterile 2-mm mesh to remove plant debris, insects, and stones. Experimental procedures were performed immediately after sample preparation. Soil microorganisms and the impact of ASFV on them were studied in hermetically sealed 10 mL glass test tubes fitted with rubber stoppers. Five grams of soil were placed at the bottom of each tube and evenly distributed. Distilled water was added to restore soil moisture to the initial level measured directly at the sampling site.

The experimental setup was based on a previously described method [13], with minor modifications. Soil moisture content corresponded to field-measured values and, due to the hermetic sealing of the tubes, remained constant throughout the experiment. Samples were maintained under a controlled light regime (approximately 12 h light/12 h dark) without aeration. At the end of the experiment, all samples were sterilized by autoclaving (121°C, 2 atm, 20 min).

### Soil physicochemical analyses

Soil pH was measured using a pH meter (Chek-Mite, Corning, Corning, NY, USA; and Checker 1, Hanna Instruments, Woonsocket, RI, USA) in a 1:2.5 mass/volume soil–water (carbon dioxide-free) suspension. In each sample, 10 g of fresh soil was used to estimate the soil moisture by drying at 105°C to constant weight.

**Table 1:** Geographic coordinates and soil pH.

Sample	GPS	pH	Location
1	40.33167625728285, 44.27391301268196	7.9–8.0	Byurakan
2	40.452311766223744, 44.64815076904667	8.5–8.6	Bjni
3	40.53371139684385, 44.72941577666204	7.3–7.4	Tsaghkadzor
4	40.40425423195387, 44.65097734092307	8.0–8.1	Charencavan
5	40.25414519796667, 44.60891718905431	8.2–8.3	Balahovit
6	40.270667994645585, 44.64020492895526	7.6–7.7	Abovyan
7	40.53845214408896, 44.775542195967724	7.2–7.3	Hrazdan
8	40.23754414746858, 44.63224116429301	7.4–7.5	Mayakovskiy
9	40.30163567493677, 44.6039541792764	7.8–7.9	Arzni
10	40.23943989242119, 44.66981322106209	7.9–8.0	Aramus
11	40.75260860531232, 44.86589156709833	7.7–7.8	Dilijan
12	40.046654815189676, 44.43697455495611	8.0–8.1	Masis
13	40.15627979223782, 44.28599564498187	8.0–8.1	Echmiadzin
14	40.172135418475825, 44.37740469399416	8.4–8.5	Musaler
15	40.156066086953786, 44.46731835597348	7.6–7.7	Yerevan
16	40.551582343289525, 44.953208171361496	7.7–7.8	Sevan
17	40.63066669671516, 43.98939753750928	8.0–8.1	Artik
18	40.81060385896342, 44.515264162080214	7.8–7.9	Vanadzor

### ASFV

The ASFV Armenia 07 virus was used in this study [14]. For all subsequent soil experiments, a working dose was prepared from a homogenate of ASFV-infected pig tissue, and the virus titer was determined in primary porcine alveolar macrophage (PAM) cultures [15]. After 1 h of adsorption at 37°C, the infected cell monolayer was washed twice to remove unbound viruses. Afterward, complete medium was added, and the samples were incubated for the indicated analysis time. The virus was collected after 72 h post-infection.

The viral titers were determined in all investigated samples using the hemadsorption microtest at HADU, as described previously [16, 17], and expressed as log10 HADU50/mL. A working dose of 10^4^ HADU was used in the experiments. In parallel, viral genome copies were measured by qPCR.

Soil samples were randomly assigned to groups: control raw soil sample; ASFV-inoculated soil (10^4^ HAD50/mL, ASFV Arm07); iASFV; and cells treated with infection buffer only (mock-infected control), which served as a negative control. Inactivated ASFV was obtained by heat treatment at 60°C for 1 h. Successful inactivation was confirmed by the absence of viral replication in PAM cultures in three independent assays.

### Soil viral DNA and RNA extraction, cDNA synthesis

Soil viruses are increasingly recognized as key regulators of microbial communities and biogeochemical cycles [18]. Therefore, viral nucleic acids were assessed to characterize the viral component of the soil ecosystem and its potential contribution to total biomass and community dynamics. The effect of ASFV on soil viral communities was investigated by extracting DNA and RNA from all soil samples.

From each sample, ground soil (0.5 g) was used to extract DNA and RNA. First, the samples were dissolved in distilled water at a ratio of 1:10. Total RNA/DNA from homogenized samples was isolated using the HiGene™ Viral RNA/DNA Prep Kit (BIOFACT, Yuseong-gu, Daejeon, Republic of Korea, Cat. No. 101-100), following the manufacturer’s instructions. All RNA/DNA samples were treated with DNase (Thermo Fisher Scientific Inc., Waltham, Massachusetts, USA) to remove genomic DNA contamination for transcriptomic analysis. RNA/DNA samples were then reverse transcribed with FIREScript® RT cDNA synthesis Kit (Solis Biodyne, Tartu, Estonia).

A NanoDrop spectrophotometer (Thermo NanoDrop 1000 Spectrophotometer, NanoDrop® ND-1000 UV-Vis, v3.8) was used to determine the concentration and quality of extracted DNA. A260/280 values were acceptable. A ratio of approximately 1.8 is considered “pure” for DNA, whereas a ratio of approximately 2.0 is considered “pure” for RNA. Purity assessment was performed to ensure the suitability and reliability of downstream qPCR analysis.

### Soil protein content

Total soil proteins provide a comprehensive picture of the physiological and functional state of the soil community. Soil protein content was measured according to a previously described method [19], with slight modifications as described by Hurisso *et al*. [20]. Modifications included increasing the centrifugation force to 3,100 × *g* for 15 minutes to ensure a clearer supernatant, and using pre-autoclaved, sterile tubes to prevent microbial contamination.

and by adjusting the soil-to-extractant ratio to 1:8 using 1.0 g of soil in 8 mL of sodium citrate to optimize protein yield for these specific soil types.

Autoclaved sterile centrifuge tubes were used. Eight milliliters of 0.02 mol L^−1^ sodium citrate (pH 7.0) was added to each tube containing 1.0 g soil sample, and the mixture was centrifuged at 3,100 × g for 15 min.

The supernatants were decanted and stored at 4°C until analysis. Protein measurements in extracts were made using the Bradford assay (Bio-Rad Laboratories, Hercules, California, USA). Five microliters of each sample extract was pipetted into individual wells of a 96-well microtiter plate containing 195 μL phosphate-buffered saline. Then, 50 μL of undiluted Bradford reagent (Bio-Rad G-250 dye) was added to each well. Plates were read 5 min later at 590 nm using a Packard SpectraCount colorimetric microplate reader (Packard Instrument Co., Meriden, Connecticut, USA). Protein concentration in each sample was calculated by comparing absorbance values to a standard curve of 0–500 μg mL^−1^ bovine serum albumin in 0.02 mol L^−1^ sodium citrate (pH 7.0) and diluted in phosphate-buffered saline.

### Quantification of cultivable bacteria and fungi

Enumeration of viable soil bacteria and fungi was performed using the plate count technique [21] on Tryptone Soya Agar (Oxoid, Basingstoke, UK, Code: CM0131) and Sabouraud Dextrose Agar (Oxoid, Code: CM0041), respectively. The prevalence of Enterobacteriaceae in soil samples was assessed using a selective medium, MacConkey agar (Oxoid MacConkey Agar No. 3, Code: CM0115).

Briefly, soil samples were passed through a 2-mm sieve and thoroughly mixed. A 1 g portion of each soil sample was diluted tenfold with sterile tap water, placed on a mechanical shaker, and shaken vigorously for 10 min before aliquots were removed. Next, a series of 10-fold dilutions was prepared, and aliquots (0.1 mL from different dilutions) were transferred and spread onto triplicate agar plates. Thus, each sowing was replicated 3 times, and no significant differences were observed. All plates were incubated under aerobic conditions at 25°C for 5 days. The number of CFUs per gram of dry soil was then calculated.

### DO concentration

Soil oxygen availability is a primary determinant of soil microbial community composition, metabolic activity, and function, and it improves root respiration and growth, ultimately contributing to enhanced plant performance [22]. DO was measured using polarography with an oxygen electrode (Milwaukee MW600 PRO, Milwaukee Instruments Inc., Rocky Mount, North Carolina, USA) [23].

Ten milliliters of water was added to 1 g of soil, and the tubes were sealed to stop oxygen flow. The tubes were incubated for 24 h to allow aerobic organisms to consume oxygen. After the incubation period, the supernatant was collected to measure DO. The electrode was calibrated at the same temperature as the soil porewater during the experiment. The polarographic DO sensor was calibrated before measurements, as electrode sensitivity and zero offset can vary due to membrane condition, electrolyte depletion, and temperature/pH effects [24].

### Gene expression analysis by qPCR

To determine ASFV gene expression, all investigated samples were analyzed. qPCR was performed using the SYBR Green method as previously described [25, 26] on an Eco Illumina Real-Time PCR system device (Illumina Inc., San Diego, CA, USA).

Each reaction mixture (20 μL) was composed of 4 μL of 5× HOT FIREPol® EvaGreen® qPCR Mix Plus (ROX) (Solis BioDyne, Tartu, Estonia), 0.3 μL of each specific primer (initial concentration 100 pmol/μL), 4 μL of template DNA/cDNA, and 11.4 μL of ddH_2_O.

Reactions were carried out under the following conditions: polymerase activation at 95°C for 12 min; 40 cycles at 95°C for 15 s, 52°C for 30 s, and 72°C for 30 s. For melt curve analysis, the following conditions were used: 65°C to 95°C, increment 0.5°C, 5 s.

For the standard curve method, PAM culture infected with ASFV was used [27, 28]. Standard curves were created using serial 10-fold dilutions of ASFV DNA. Quantitative analysis of DNA/cDNA copies was performed by comparison with known numbers of ASFV genome units.

The fluorescence threshold value (Ct) was calculated using the ECO-Illumina system software (v 5.0). To align the cDNA plots, Cq values were rescaled after comparison with viral genome copy numbers and converted to absolute values on the y-axis for better visualization. Amplification efficiency and specificity were assessed by analyzing the standard curve correlation coefficient (R² value) and melt curve profiles. Values ≥ 0.99 were considered acceptable.

Primers used for amplification were designed based on FASTA sequences retrieved from the NCBI GenBank database. All primers were synthesized by Integrated DNA Technologies (IDT, Coralville, Iowa, USA).

**Table 2:** Primer details used in the study.

Virus/gene	Sequence	Temperature (°C)	Amplicon size	GenBank
ASFV thymidine kinase gene K196R	F: GCAGTTGTCGTAGATGAAG R: CGAAGGAAGCATTGAGTC	53.58 / 53.17	109 bp	FR682468.2
Megavirus chiliensis mg9	F: CTCTTAAATCTTTCACCCTACC R: CAGCACATCTTGGAACAC	53.92 / 53.68	169 bp	NC_016072.1, JN258408.2
Mimivirus RNases H gene	F: CAGATTCTACTTACAGTGTCAATA R: GACCAGTATGTGCTTCAAC	53.93 / 53.72	170 bp	AY653733.1, HQ336222.2, JN036606.1, JF801956.1, KM982403.1, KT599914.1, KF959826.2, KU761889.1
Phycodnavirus DNA polymerase	F: GAGTCTGTATCCGAGTATCA R: TTGTCAAACTTATTCTCCATCA	53.15 / 53.38	78 bp	HM629733.1

### Statistical analysis

All experiments were conducted in triplicate. Control sample, inactive virus, and virus-treated samples were processed, extracted, and analyzed independently. The significance of virus-induced changes was evaluated by two-tailed Student’s t-test for parametric values and Mann–Whitney U-test for non-parametric values; p < 0.05 was considered significant.

## RESULTS

### Dynamics of ASFV in soil

**Figure 2:**
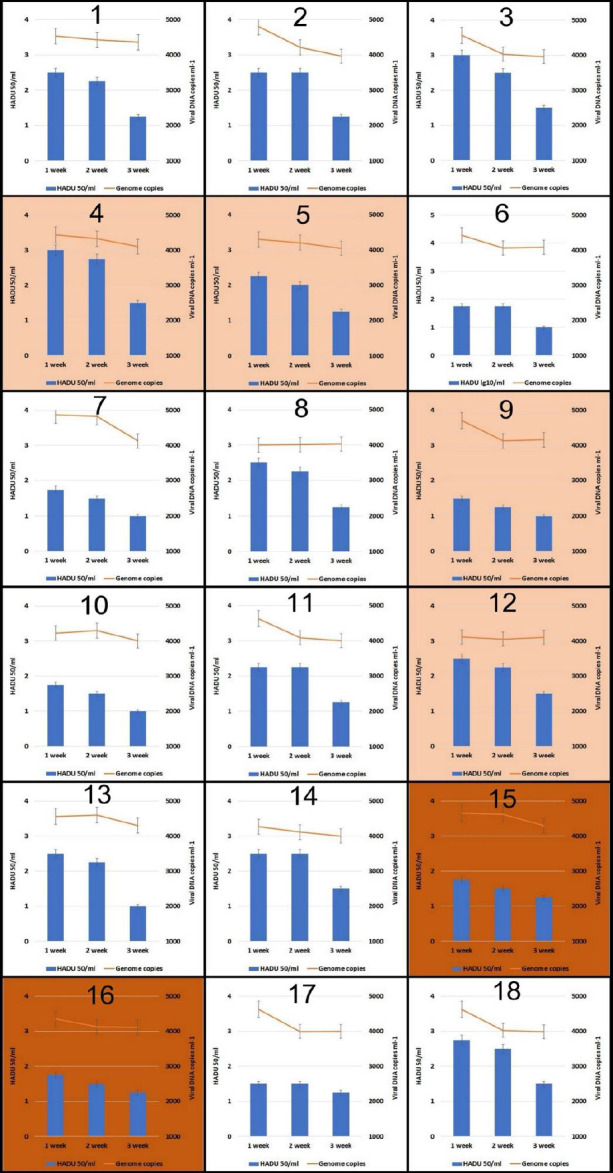
African swine fever virus levels (measured by qPCR and HADU) and transcriptional activity in soil samples during 3-week incubation at 24°C–26°C. Color intensity represents the level of transcriptional activity (no color = no transcriptional activity; light orange = low transcriptional activity; dark orange = high transcriptional activity).

A comprehensive analysis of ASFV status was performed, including assessment of infectious particle content, genome copy numbers, and transcriptional activity, as well as their dynamics in soil samples. Figure 2 illustrates the quantitative changes in ASFV during the experimental period. Notably, transcriptional activity of viral genes was detected in several soil samples (4, 5, 9, 12, and 15–16).

The quantitative Cq values obtained from cDNA exceeded the corresponding DNA-derived values by approximately 10–15% and, in some cases, by up to 80–90%. Importantly, viral titers showed a consistent decline by the end of the experiment. This observation suggests that the virus may have entered certain non-susceptible organisms; however, no evidence of complete genome replication was detected during the experimental period.

### Impact of ASFV on nucleic acid levels in soil

**Figure 3:**
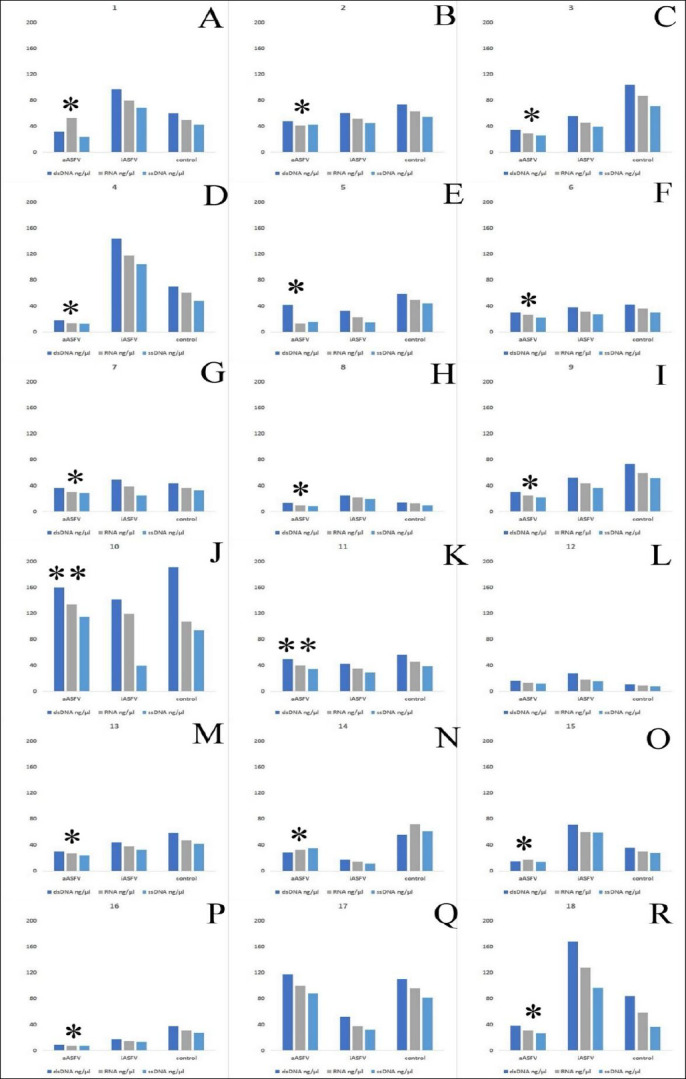
Short-length, predominantly viral-origin dsDNA, RNA, and ssDNA concentrations (ng/μL) in soil samples with active ASFV (aASFV), inactive ASFV (iASFV), and control samples after the 1st week of incubation at 24°C–26°C. A = 1st sample, B = 2nd sample, C = 3rd sample, D = 4th sample, E = 5th sample, F = 6th sample, G = 7th sample, H = 8th sample, I = 9th sample, J = 10th sample, K = 11th sample, L = 12th sample, M = 13th sample, N = 14th sample, O = 15th sample, P = 16th sample, Q = 17th sample, R = 18th sample. *Significant difference by Mann–Whitney U test (p < 0.05–0.001) compared with control and/or iASFV. **Significant difference by Mann–Whitney U test (p < 0.05) in dsDNA concentration compared with control.

Given that the kit is designed for quantitative analysis of DNA and RNA in soil, but does not ensure complete specificity, the obtained data are interpreted as mainly reflecting viral nucleic acids. Figure 3 presents data on the concentrations of dsDNA, ssDNA, and RNA in soil samples one week after incubation with ASFV.

As shown in Figure 3, the majority of soil samples exhibited a significant reduction in the levels of these nucleic acids. The exception is samples 10, 11, 12, and 17, where the content of viral nucleic acids a week after the start of the experiment exceeded or equaled the control values.

### Assessment of soil protein content following ASFV contamination

Active ASFV led to a decrease in total protein content in most of the soil samples studied. Exceptions were observed in samples 9, 11, and 17, where protein content increased. By the end of the experiment (week 3), the differences between the control and experimental groups tended to diminish, with no significant changes observed (data not shown) (Table S1).

### Impact of ASFV on soil bacterial and fungal communities

Changes in the total number of cultivable bacteria and fungi in the soil samples in the presence of ASFV are summarized in Table S2. The most common trend was an increase in the total number of cultivable bacteria and fungi in the presence of aASFV, compared with the control soil sample (72.2%).

**Figure 4:**
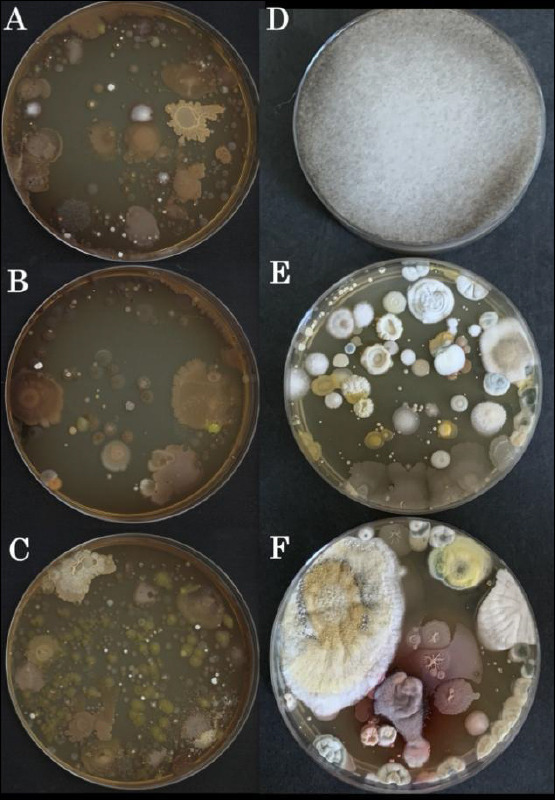
Growth of colonies from soil samples on agar plates. Control samples (A, D); samples incubated with inactivated ASFV (B, E); samples infected with active ASFV (C, F). (A–C) Sample 14 on Tryptone Soya Agar. (D–F) Sample 9 on Sabouraud Dextrose Agar.

The impact of ASFV on bacterial diversity varied among the soil samples. Notably, compared to the control, bacterial diversity decreased in the presence of iASFV and aASFV (61.1% and 44.4% of samples, respectively). Figure 4 illustrates the main strategies for changing the biomass and biodiversity in soil samples under the influence of ASFV.

Noticeably, compared with the control (Figure 4A), there was an increase in the number of colonies under the influence of iASFV (Figure 4B) and aASFV (Figure 4C).

The diversity of fungal colonies was not affected by the presence of ASFV in 50% of soil samples (Figure 4D–F). Figure 4D–F illustrates the main strategy for changing the biomass and diversity in soil samples under the influence of ASFV. Figure 4D shows a fungal colony on the control plate of soil sample 9, demonstrating how one fungal species took over the entire culture medium and suppressed the remaining fungal species (“winner” dominance). Figure 4E and Figure 4F (iASFV and aASFV, respectively) show the development of multiple fungal colonies in the absence of a dominant “winner.”

### Effects of ASFV on soil biomass, biodiversity, and oxygen dynamics

**Figure 5:**
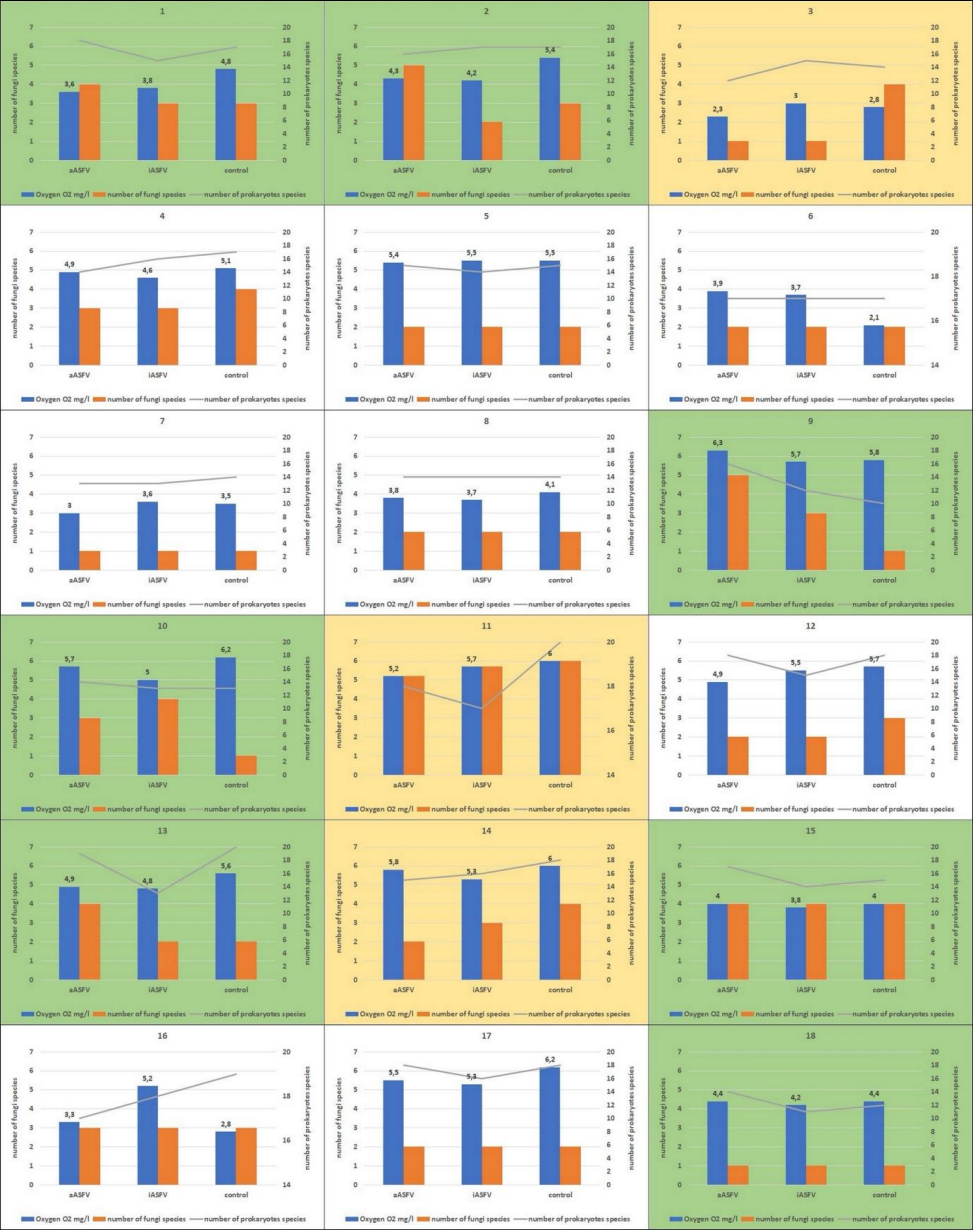
Changes in oxygen levels and species diversity in soil samples under the influence of African swine fever virus. 1 = 1st sample, 2 = 2nd sample,…, 18 = 18th sample. Green = increased biodiversity; Yellow = decreased biodiversity; No color = no significant difference compared to control. *Significant difference by Mann–Whitney U test (p < 0.05–0.001) compared with control and/or iASFV. **Significant difference by Mann–Whitney U test (p < 0.05).

Figure 5 illustrates the main strategies for changing the biomass and biodiversity in soil samples under the influence of ASFV. From Figure 5, it follows that in most samples, the presence of ASFV leads to either an increase in the number of species (prokaryotes and/or fungi) or no change. The O_2_ content is inversely proportional to the number of prokaryotic species.

### Impact of active ASFV on soil giant viruses

Viruses similar in genome to the Megavirus chiliensis were found earlier in water samples contaminated with sewage waters in some settlements of Armenia (unpublished data). However, both megaviruses and picodnaviruses are detected for the first time in soil samples in Armenia (possibly these soil samples were contaminated with sewage water).

Giant viruses in soil are now considered influential players in ecosystem processes due to their ability to infect a broad spectrum of eukaryotic organisms. Analyses of medium- to high-quality giant virus genomes have revealed both a core set of shared functional traits and previously unrecognized auxiliary metabolic genes involved in carbon, sulfur, and phosphorus metabolism [29]. In this context, it is important to study how ASFV affects giant viruses.

The presence of ASFV reduced quantitative indicators of both megaviruses and picodnaviruses in soil samples. However, the number of soil samples positive for these viruses was too small (2 out of 18) to draw reliable conclusions (Table S3).

It should be noted that in both samples with identified megaviruses, their disappearance or a sharp decrease in content was noted, which coincided with the decrease in the total concentrations of viral nucleic acids in most soil samples after 1 week of incubation with aASFV.

### Correlation analysis of the main investigated indices

A correlation matrix was generated to explore relationships among protein content, nucleic acid levels (DNA/RNA), microbial populations, and environmental factors in soil samples exposed to ASFV (Table S4).

Protein content was found to correlate positively and significantly with DNA/RNA levels (r = 0.466, p < 0.05), CFUs of fungi (r = 0.481, p < 0.05), and CFUs of enterobacteria (r = 0.454, p < 0.05).

A significant negative correlation was observed between DNA/RNA content and O_2_ levels (r = −0.456, p < 0.05). Giant virus abundance exhibited a significant negative correlation with enterobacterial CFUs (r = −0.457, p < 0.05). O_2_ content was also inversely correlated with CFUs of enterobacteria (r = −0.578, p < 0.01).

Notably, a strong positive correlation was found between fungal and prokaryotic abundance (r = 0.856, p < 0.01). Total culturable microbial counts showed a significant positive correlation with CFUs of fungi (r = 0.502, p < 0.05).

## DISCUSSION

### ASFV and soil ecosystem interactions

ASFV is a highly stable and environmentally persistent pathogen capable of entering soil ecosystems through biological contamination [7]. Although its transmission pathways and environmental persistence are well documented, its ecological impact, particularly on soil microbial biomass and biodiversity within the main microbial community, remains poorly understood. This study focused on two fundamental indicators of soil ecosystem health: biomass, assessed via protein content and nucleic acid concentrations; and biodiversity, evaluated through the composition of microbial (bacterial and fungal) and viral populations.

The quantified nucleic acids (DNA and RNA) were interpreted primarily as viral in origin because the extraction kits were optimized for viral nucleic acid recovery; however, co-extraction of non-viral nucleic acids cannot be excluded. Therefore, these results should be considered preliminary and highlight a promising direction for future investigations of the soil virome and ASFV-associated viral dynamics.

### Role of giant viruses and microbial interactions

Relatively recently, giant viruses have been recognized as a persistent component of soil ecosystems, where they may contribute to biogeochemical cycling of carbon and phosphorus [29, 30]. In the present study, the abundance of giant viruses in soil samples did not show significant changes in the presence of ASFV.

Interestingly, the abundance of giant viruses exhibited a significant negative correlation with enterobacterial CFUs (r = −0.457, p < 0.05), suggesting a potential ecological interaction between these viral taxa and specific bacterial groups. One possible explanation is linked to the ecological role of giant viruses, which often infect protozoa that graze on prokaryotes, including Enterobacteriaceae. A reduction in protozoan populations could theoretically result in decreased giant virus abundance and a concurrent increase in bacterial populations, including Enterobacteriaceae.

### Effects of ASFV on biomass indicators

Analysis of ASFV influence on biomass indicators revealed notable trends. Protein content was significantly and positively correlated with DNA/RNA levels (r = 0.466, p < 0.05), fungal CFUs (r = 0.481, p < 0.05), and enterobacterial CFUs (r = 0.454, p < 0.05). These correlations suggest that higher microbial biomass supports a more substantial viral presence, likely through increased availability of host cells suitable for viral replication or maintenance. This is consistent with findings by Matz *et al*. [31], which indicate that direct protein measurements can reliably estimate microbial biomass.

The study also identified a significant presence of eukaryotic viruses in the soil, particularly RNA viruses, which are likely to influence interspecies interactions within the microbial community [32]. Changes in viral abundance may thus reshape the composition of the soil ecosystem.

### ASFV-induced reductions in nucleic acids and potential mechanisms

Notably, ASFV exposure was associated with reduced DNA and RNA levels in most soil samples, suggesting a decline in viral biomass. One plausible explanation is ASFV-induced cellular stress or apoptosis in microbial hosts, thereby reducing the pool of viable host cells available to other viruses, although this mechanism remains hypothetical and requires direct experimental validation.

It is known that even in the absence of productive infection, viral capsid components can trigger apoptosis in non-susceptible cells [33–35], suggesting a possible mechanism that could contribute to systemic reductions in host biomass and, indirectly, in viral abundance.

Furthermore, a negative correlation between oxygen levels and DNA/RNA was observed (r = −0.456, p < 0.05). This may reflect reduced microbial respiration due to ASFV-induced mortality, leading to lower oxygen consumption and an increase in soil oxygen availability [36, 37]. Thus, higher oxygen concentrations could be a consequence rather than a cause of suppressed microbial activity under the influence of ASFV.

### Divergence between biomass and biodiversity patterns

Interestingly, biodiversity trends did not parallel changes in biomass. While both total protein and nucleic acid levels declined, microbial diversity appeared to increase, particularly among fungal and prokaryotic populations. A strong positive correlation between fungal and prokaryotic CFUs (r = 0.856, p < 0.01) suggests possible synergistic interactions or co-resilience mechanisms in response to viral pressure.

Similar patterns of increased diversity concurrent with decreased biomass have been reported in other ecosystems, such as the gut microbiome during hepatitis B progression [23].

### Ecological mechanisms and theoretical implications

**Scheme 1:**
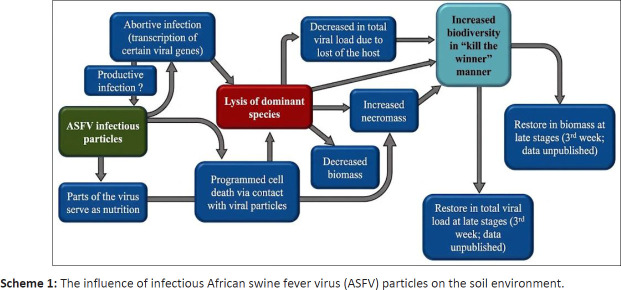
The influence of infectious African swine fever virus (ASFV) particles on the soil environment.

In simplified form, the interaction between ASFV and the soil-living community is presented in Scheme 1. The influence of viruses in terrestrial ecosystems may be ecologically analogous to their roles in marine environments. Trubl et al. [38] identify three principal virus–host interaction pathways: (1) lysis of host cells, (2) alteration of host metabolism, and (3) virus-mediated horizontal gene transfer.

Given the absence of detectable ASFV replication, the first two pathways are more relevant in this context. These findings support the hypothesis that viral presence, especially that of non-replicating viruses, can act as a selective force, reshaping community structure by suppressing dominant taxa and enabling the proliferation of less competitive or stress-tolerant organisms. This phenomenon aligns conceptually with the “Kill the Winner” model [39], in which viruses preferentially infect the most active microbial taxa, thereby maintaining community diversity.

### Non-replicative ecological effects of ASFV

While no increase in ASFV titers was detected in any of the samples relative to controls, suggesting limited or no productive replication, ecological effects were nonetheless observed. Consequently, these effects may be mediated through non-replicative mechanisms, such as immune stimulation, capsid-mediated cellular stress, or apoptosis, rather than active viral propagation, although direct mechanistic evidence remains to be established.

This interpretation is further supported by the absence of correlation between ASFV K196R gene expression and other measured indicators. These findings are consistent with emerging perspectives that viruses, regardless of replication status, play active roles in ecosystem regulation, modulating everything from host physiology to community composition and nutrient flux [40].

### Study limitations and future perspectives

Taken together, the findings suggest that ASFV presence in soil environments may be associated with measurable ecological shifts in microbial biomass and community structure, even in the absence of detectable viral replication. These observations extend the current understanding of ASFV ecology beyond persistence and transmission, highlighting the importance of considering environmental interactions between viruses and soil microbial communities.

While the mechanisms underlying these patterns remain unresolved, the observed associations raise important questions regarding potential indirect effects of viral particles on microbial dynamics and ecosystem functioning. From a broader perspective, virus-associated changes in microbial communities may influence soil ecosystem services, including nutrient cycling and microbial-mediated carbon and phosphorus turnover, thereby linking animal disease dynamics with environmental health within a One Health framework.

At the same time, the present study has several limitations, including reliance on culturable microorganisms and targeted molecular assays, limited detection of giant viruses, and restricted generalizability beyond Armenian anthrosols. Future studies integrating metagenomic approaches, expanded environmental characterization, and comparative experimental designs will be necessary to clarify underlying mechanisms and evaluate the ecological significance of ASFV–soil interactions.

Thus, this work provides an initial ecological framework and highlights a promising direction for future interdisciplinary research exploring the environmental dimensions of ASFV biology.

## CONCLUSION

The present study demonstrates that ASFV exerts complex, multidirectional effects on soil ecosystems, characterized by a consistent reduction in total microbial biomass and an increase in microbial diversity. The observed decline in protein content and viral nucleic acids across most samples indicates a suppression of overall biomass, whereas the concurrent increase in CFU of bacteria and fungi suggests a restructuring of the microbial community. The detection of transcriptional activity without evidence of complete viral replication further supports the hypothesis that ASFV influences soil systems primarily through indirect or non-replicative mechanisms.

From a practical perspective, these findings highlight the potential environmental implications of ASFV contamination beyond its established role as a livestock pathogen. Changes in microbial biomass and diversity may affect key soil functions, including nutrient cycling, organic matter decomposition, and overall soil health. This has important implications for agricultural systems, particularly in regions affected by ASF outbreaks, where soil-mediated ecological changes could impact productivity and ecosystem stability.

A major strength of this study lies in its integrative approach, combining physicochemical analysis, qPCR-based quantification of nucleic acids, microbial culturing, and DO measurements to provide a comprehensive assessment of ASFV–soil interactions. Additionally, the use of both aASFV and iASFV allowed differentiation between active and passive viral effects. However, several limitations should be acknowledged. The reliance on culturable microorganisms may underestimate total microbial diversity, and the specificity of viral nucleic acid extraction does not fully exclude non-viral contributions. The limited detection of giant viruses and the focus on a specific soil type (anthrosols) further restrict the generalizability of the findings.

Future research should incorporate metagenomic and metatranscriptomic approaches to better resolve microbial and viral community dynamics. Expanded studies across diverse soil types and environmental conditions are needed to validate these findings and elucidate underlying mechanisms. Investigations into the long-term ecological consequences of ASFV persistence and its interactions with non-host organisms will be particularly valuable.

In conclusion, ASFV represents not only a significant veterinary threat but also a potential ecological modulator within soil environments. The study provides initial evidence that ASFV can alter microbial biomass and biodiversity in soil systems, emphasizing the need to integrate environmental considerations into ASFV research within a One Health framework.

## DATA AVAILABILITY

The data generated during the study are included in the manuscript.

The supplementary data can be made available from the corresponding author upon request.

## AUTHORS’ CONTRIBUTIONS

ZK and AS: Conceptualization, data curation, project administration, supervision, validation, visualization, writing – original draft. MZ, SH, AA, SHo, NB, HA, VG, TV, BB, AK, AP,HAv, EK: Formal analysis. MZ, SH, AA, SHo, NB, HA, VG, TV, BB, AK, LH, AP, LA, EK, HV, EA, and HAv: Investigation. AS, AA, EA, ShH, SH, AP, and AH: Methodology. AK, VG, ZK, and AS: Software. AP, SH, and HAv: Writing – review and editing. All authors have read and approved the final version of the manuscript.

## References

[1] Oura CA, Powell PP, Anderson E, Parkhouse RM (1998). The pathogenesis of African swine fever in the resistant bushpig. J Gen Virol.

[2] Salguero FJ (2020). Comparative Pathology and Pathogenesis of African Swine Fever Infection in Swine. Front Vet Sci.

[3] Gryz J, Krauze-Gryz D (2019). Indirect Influence of African Swine Fever Outbreak on the Raven (Corvus corax) Population. Animals.

[4] Hakobyan SA, Ross PA, Bayramyan NV, Poghosyan AA, Avetisyan AS, Avagyan HR (2022). Experimental models of ecological niches for African swine fever virus. Vet Microbiol.

[5] Anand U, Bianco F, Suresh S, Tripathi V, Núñez-Delgado A, Race M (2021). SARS-CoV-2 and other viruses in soil: An environmental outlook. Environ Res.

[6] Chenais E, Depner K, Guberti V, Dietze K, Viltrop A, Stahl K (2019). Epidemiological considerations on African swine fever in Europe 2014–2018. Porc Health Manag.

[7] Carlson J, Fischer M, Zani L, Eschbaumer M, Fuchs W, Mettenleiter T (2020). Stability of African Swine Fever Virus in Soil and Options to Mitigate the Potential Transmission Risk. Pathogens.

[8] Prodelalova J, Kavanova L, Salat J, Moutelikova R, Kobzova S, Krasna M (2022). Experimental evidence of the long-term survival of infective African swine fever virus strain Ba71V in soil under different conditions. Pathogens.

[9] Tanneberger F, Abd El Wahed A, Fischer M, Blome S, Truyen U (2021). The efficacy of disinfection on modified vaccinia Ankara and African swine fever virus in various forest soil types. Viruses.

[10] Tanneberger F, Abd El Wahed A, Fischer M, Deutschmann P, Roszyk H, Carrau T (2022). Efficacy of liming forest soil in the context of African swine fever virus. Viruses.

[11] Liu Z, Huang Y, Zhang T, Meng D, Jiang Z, Yang Z (2023). Viruses regulate microbial community assembly together with environmental factors in acid mine drainage. Appl Environ Microbiol.

[12] Huang X, Braga LPP, Ding C, Yang B, Ge T, Di H (2024). Impact of viruses on prokaryotic communities and greenhouse gas emissions in agricultural soils. Adv Sci.

[13] Bosco F, Mollea C (2021). Biodegradation of Natural Rubber: Microcosm Study. Water Air Soil Pollut.

[14] Rowlands RJ, Michaud V, Heath L, Hutchings G, Oura C, Vosloo W (2008). African swine fever virus isolate, Georgia, 2007. Emerg Infect Dis.

[15] Forman AJ, Wardley RC, Norley SG (1983). Interactions of porcine alveolar macrophages and bone marrow cells with African swine fever virus and virus-infected cells. Vet Microbiol.

[16] Enjuanes L, Carrascosa AL, Moreno MA, Viñuela E (1976). Titration of African swine fever (ASF) virus. J Gen Virol.

[17] Carrascosa AL, Bustos MJ, de Leon P (2011). Methods for growing and titrating African swine fever virus: Field and laboratory samples. Curr Protoc Cell Biol.

[18] Xiao J, Liu C, Wei R, Chi Z, Zhang P, Yu Z (2025). Fertilization Strategies Regulate Soil Viral Diversity and Functional Potentials in Nutrient Cycling. Agronomy.

[19] Valsange A, Evarkar SP, Tawde SV, Kareppa BM, Gujar RS (2012). Extraction, purification and analysis of thermal stability of xylose isomerase. Current Botany.

[20] Hurisso TT, Moebius-Clune DJ, Culman SW, Moebius-Clune BN, Thies JE, van Es HM (2018). Soil protein as a rapid soil health indicator of potentially available organic nitrogen. Agric Environ Lett.

[21] Carter MR, Gregorich EG (2007). Soil sampling and methods of analysis.

[22] Sun M, Liu X, Shi K, Peng F, Xiao Y (2022). Effects of Root Zone Aeration on Soil Microbes Species in a Peach Tree Rhizosphere and Root Growth. Microorganisms.

[23] Chen Y, Chen Z, Guo R, Chen N, Lu H, Huang S (2011). Correlation between gastrointestinal fungi and varying degrees of chronic hepatitis B virus infection. Diagn Microbiol Infect Dis.

[24] Johnson CD, Paul DW (2005). In situ calibrated oxygen electrode. Sens Actuators B Chem.

[25] Ginzinger DG (2002). Gene quantification using real-time quantitative PCR: An emerging technology hits the mainstream. Exp Hematol.

[26] Yin JL, Shackel NA, Zekry A, McGuinness PH, Richards C, van Putten K (2001). Real-time reverse transcriptase-polymerase chain reaction (RT-PCR) for measurement of cytokine and growth factor mRNA expression with fluorogenic probes or SYBR Green I. Immunol Cell Biol.

[27] Poghosyan A, Hakobyan S, Avagyan H, Avetisyan A, Bayramyan N, Hakobyan L (2024). The role of gastropods in African swine fever virus ecology. Virol J.

[28] Avagyan HR, Hakobyan SA, Poghosyan AA, Bayramyan NV, Arzumanyan HH, Abroyan LO (2022). African Swine Fever Virus Manipulates the Cell Cycle of G0-Infected Cells to Access Cellular Nucleotides. Viruses.

[29] Liang JL, Feng SW, Jia P, Lu JL, Yi X, Gao SM (2024). Unraveling the habitat preferences, ecological drivers, potential hosts, and auxiliary metabolism of soil giant viruses across China. Microbiome.

[30] Schulz F, Alteio L, Goudeau D, Ryan EM, Yu FB, Malmstrom RR (2018). Hidden diversity of soil giant viruses. Nat Commun.

[31] Matz C, Jürgens K (2003). Interaction of nutrient limitation and protozoan grazing determines the phenotypic structure of a bacterial community. Microb Ecol.

[32] Hillary LS, Adriaenssens EM, Jones DL, McDonald JE (2022). RNA-viromics reveals diverse communities of soil RNA viruses with the potential to affect grassland ecosystems across multiple trophic levels. ISME Commun.

[33] Ivanovska I, Hardwick JM (2005). Viruses activate a genetically conserved cell death pathway in a unicellular organism. J Cell Biol.

[34] Robson GD (2006). Programmed cell death in the aspergilli and other filamentous fungi. Med Mycol.

[35] Ramsdale M (2008). Programmed cell death in pathogenic fungi. Biochim Biophys Acta.

[36] Wu Y, Cai P, Jing X, Niu X, Ji D, Ashry NM (2019). Soil biofilm formation enhances microbial community diversity and metabolic activity. Environ Int.

[37] Yan H, Yang F, Gao J, Peng Z, Chen W (2019). Subsoil microbial community responses to air exposure and legume growth depend on soil properties across different depths. Sci Rep.

[38] Trubl G, Hyman P, Roux S, Abedon ST (2020). Coming-of-age characterization of soil viruses: A user's guide to virus isolation, detection within metagenomes, and viromics. Soil Syst.

[39] Thingstad TF (2000). Elements of a theory for the mechanisms controlling abundance, diversity, and biogeochemical role of lytic bacterial viruses in aquatic systems. Limnol Oceanogr.

[40] Hamblin SR, White PA, Tanaka MM (2014). Viral niche construction alters hosts and ecosystems at multiple scales. Trends Ecol Evol.

